# The impact of agar depth on antimicrobial susceptibility testing by disc diffusion

**DOI:** 10.1099/jmm.0.001890

**Published:** 2024-09-18

**Authors:** Ioana D. Olaru, Sarah Schoeler, Frieder Schaumburg

**Affiliations:** 1 Institute of Medical Microbiology, University Hospital Münster, Münster, Germany

**Keywords:** antimicrobial susceptibility testing, AST, disc diffusion

## Abstract

**Introduction.** The European Committee on Antimicrobial Susceptibility Testing (EUCAST) specifies the agar depth (4±0.5 mm) when performing antimicrobial susceptibility testing (AST). Since the infrastructure to produce standardized agar may be lacking in settings with limited resources, we wanted to examine to what extent variation in agar depth affects the inhibition zone diameters of quality control (QC) strains and AST of clinical isolates.

**Methods.** The inhibition zone diameters on Mueller–Hinton II agar with different depths (2–6 mm) were tested for various QC strain–antimicrobial agent combinations using *Escherichia coli* ATCC 25922, *Pseudomonas aeruginosa* ATCC 27853 and *Staphylococcus aureus* ATCC 29213. The relationship between zone diameters at different agar depths and MICs was investigated for 35 clinical isolates (*E. coli*, *Klebsiella pneumoniae*, *S. aureus* and *P. aeruginosa*) from Sierra Leone using MICs as the reference.

**Results.** The inhibition zone diameters were within the acceptance ranges as defined by the EUCAST for the majority of QC strains and antimicrobials, independent of the agar depth. At extreme agar depths, inhibition zones were more frequently out of range. The accuracy of AST varied for clinical isolates at different agar depths for categorical agreement (85.8–94.6%), major error rate (0.4–2.1%) and very major error rate (VME: 3.3–12.5%).

**Conclusions.** Even if the QC strains were in the acceptance range at different agar depths, this does not rule out unacceptably high VME rates (>3%) in clinical isolates.

## Background

Reliable antimicrobial susceptibility testing (AST) results are key to an effective antimicrobial therapy. For in-house prepared media, several conditions have to be met to ensure high-quality AST results (e.g. incubation time, agar depth of 4±0.5 mm and pH) [1]. In low-resource settings, it can be challenging to meet all test specifications. Deviation from these specifications may affect AST accuracy and ultimately patient outcomes.

Since the early days of disc diffusion, it has been known that agar depth affects AST: the inhibition zones become smaller with increasing agar depth [2] because of differences in the volumes of diffusion. However, it is not clear to what extent this may be relevant when performing AST for clinical samples.

The aim of this study was to address to what extent agar depth can affect AST results. First, we wanted to determine to what extent inhibition zones of QC strains are within the target and range at different agar depths. Second, we assessed the accuracy of AST on different agar depths when using clinical isolates from a low-resource setting. The findings of this study will aid the interpretation of AST results from laboratories, which prepare media in-house without accurately controlling the depth of agar.

## Methods

### Agar preparation

Mueller–Hinton II agar (MH II, BBL Mueller–Hinton II Agar, BD, Heidelberg, Germany) was prepared in Petri dishes (82.1473, Sarstedt, Nümbrecht, Germany, with an internal diameter of 86 mm) following the manufacturer’s recommendation. Adjustment of pH was not done [3]. The volume was calculated based on the specifications of the Petri dishes. Plates for the same organism were prepared during the same session. Agar depth was not further measured. The following volumes of MH II agar were used to achieve the respective agar depth (in brackets): 12 ml (2 mm), 18 ml (3 mm), 24 ml (4 mm), 30 ml (5 mm) and 36 ml (6 mm).

### Quality control

All analyses were performed in a laboratory certified according to DIN EN ISO 15189.

The applied discs and concentrations are listed for each species in Supplementary Material (Tables S1–S3, available in the online version of this article, Oxoid, Wesel, Germany).

In the first step, AST was conducted using quality control (QC) strains of *Escherichia coli* ATCC 25922, *Pseudomonas aeruginosa* ATCC 27853 and *Staphylococcus aureus* ATCC 29213 on in-house prepared MH II media with agar depths of 2–6 mm and on a commercial MH II agar (BD; agar depth of 4 mm). Overnight cultures of *E. coli* ATCC 25922, *P. aeruginosa* ATCC 27853 and *S. aureus* ATCC 29213 were inoculated in quadruplicate and tested in parallel. Target and ranges as recommended by the European Committee on Antimicrobial Susceptibility Testing (EUCAST) were used for interpreting the results [1]. To enable comparisons between the different media (commercial vs. in-house), the mean inhibition zone diameters from the quadruplicate tests were used to calculate the penalty scores for each agar depth [3]. Briefly, scores were given as follows: 0 points if the zone was on target or within 1 mm, −1 point for zones within ±2 mm of target, −3 points if the zones were ±3 mm of target but within range and −5 points if outside the range. The total score was calculated by summing up all individual scores. The proportion of zones within ±1 of the target and that of zones out of range were also computed.

### AST of clinical isolates on different agar depths

The ascertainment of growth inhibition for different agar depths was done as suggested for the evaluation of new AST test devices [4]. Here, the in-house MH II agars with different depths were considered the new test devices.

In the second step, 35 patient isolates (10 *Klebsiella pneumoniae*, 10 *E. coli*, 10 *S*. *aureus* and 5 *P*. *aeruginosa*) were randomly selected from a collection of bacteria isolated from chronic wounds in Sierra Leone [5]. A standard set of antimicrobial agents from different classes was tested (number of agents in brackets) for *S. aureus* (*n*=6, Table S1), *P. aeruginosa* (*n*=6, Table S2), *E. coli* (*n*=8, Table S3) and *K. pneumoniae* (*n*=7, Table S3). This resulted in 240 readings per agar depth (10 *S*. *aureus*×6 agents+5 *P*. *aeruginosa*×6 agents+10 *E*. *coli*×8 agents+10 *K*. *pneumoniae*×7 agents).

An MIC-based method was chosen as a reference standard: broth microdilution (BMD, MICRONAUT, Merlin/Bruker, Bremen, Germany) for Gram-negative bacteria and Vitek2 cards (AST-P 654) for *S. aureus*. The following QC strains were used for BMD: *E. coli* ATCC 25922, *K. pneumoniae* ATCC 700603 and *P. aeruginosa* ATCC 27853. *S. aureus* ATCC 29213 was selected as the QC for Vitek2 AST.

Tests were performed and interpreted according to EUCAST clinical breakpoints (version 13.1) [6].

The category ‘I’ (susceptible to increased exposure) was classified as susceptible. The accuracy [categorical agreement (CA), major error (ME) and very major error (VME)] [7] was calculated for each agar depth, and the MIC-based methods (BMD or Vitek2) were used as reference (Table 1). Error rates were calculated using all the isolates as the denominator.

**Table 1.** Accuracy of AST on different depths of MH II agar using clinical isolates from Sierra Leone

Agar depth (mm)	CA [% (*n*/*n*_total_)]	ME [% (*n*/*n*_total_)]	VME [% (*n*/*n*_total_)]
2	85.8% (206/240)	1.7% (4/240)	12.5% (30/240)
3	92.9% (223/240)	0.4% (1/240)	6.6% (16/240)
4	93.3% (224/240)	1.3% (3/240)	5.4% (13/240)
5	93.8% (225/240)	1.3% (3/240)	5.0% (12/240)
6	94.6% (227/240)	2.1% (5/240)	3.3% (8/240)

## Ethical approval

The patient strains involved in this study were from Sierra Leone. Ethical approval was obtained from the Sierra Leone Ethics and Scientific Review Committee (version 1.0 of 12 January 2019). Patients provided a written informed consent prior to inclusion [5].

## Results

### ATCC QC strains

**Fig. 1.**
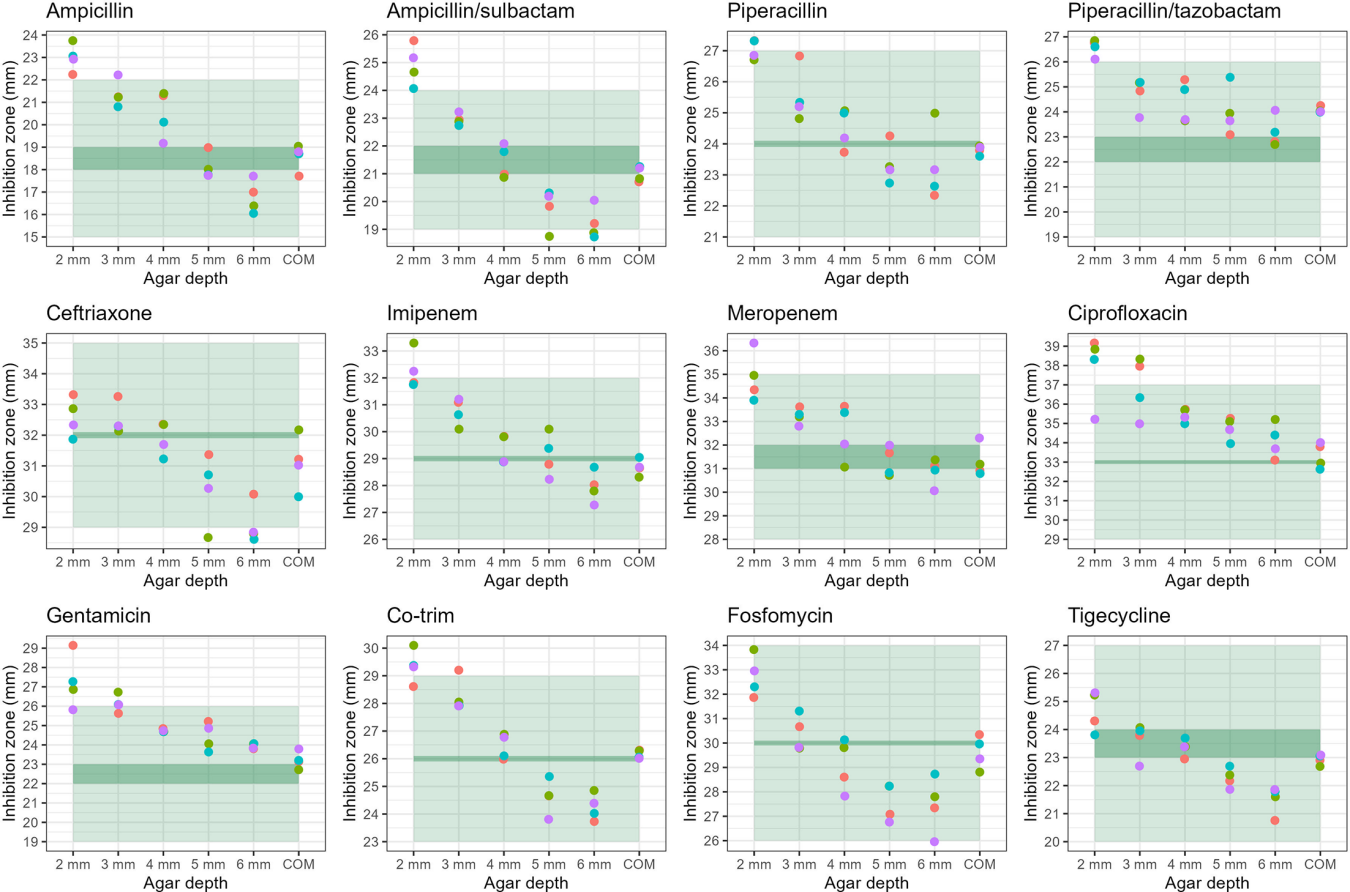
Comparison of *E. coli* ATCC 25922 inhibition zone diameters for the five different agar depths of in-house MH agar and a commercial MH agar (COM) according to target (dark green) and range (light green).

When using QC strains, the zones of inhibition for the different depths of in-house agar were frequently within ranges except for 2-mm agar depth (Tables S5–S8, Figs1^3^). At the optimal agar depth of 4 mm, the zones of inhibition were always within range. Between 70 and 75% of tests (depending on the bacterial species) were within 1 mm of the target inhibition zone, while the commercial MH media were almost always within 1 mm of the target (Table S8). At extreme agar depths, the inhibition zones were frequently off-target with 27–30% out of range for 2-mm depth and 0–60% for 6 mm (Table S8). For all organisms and antimicrobials, the inhibition zones decreased with the increase in agar depth.

**Fig. 2.**
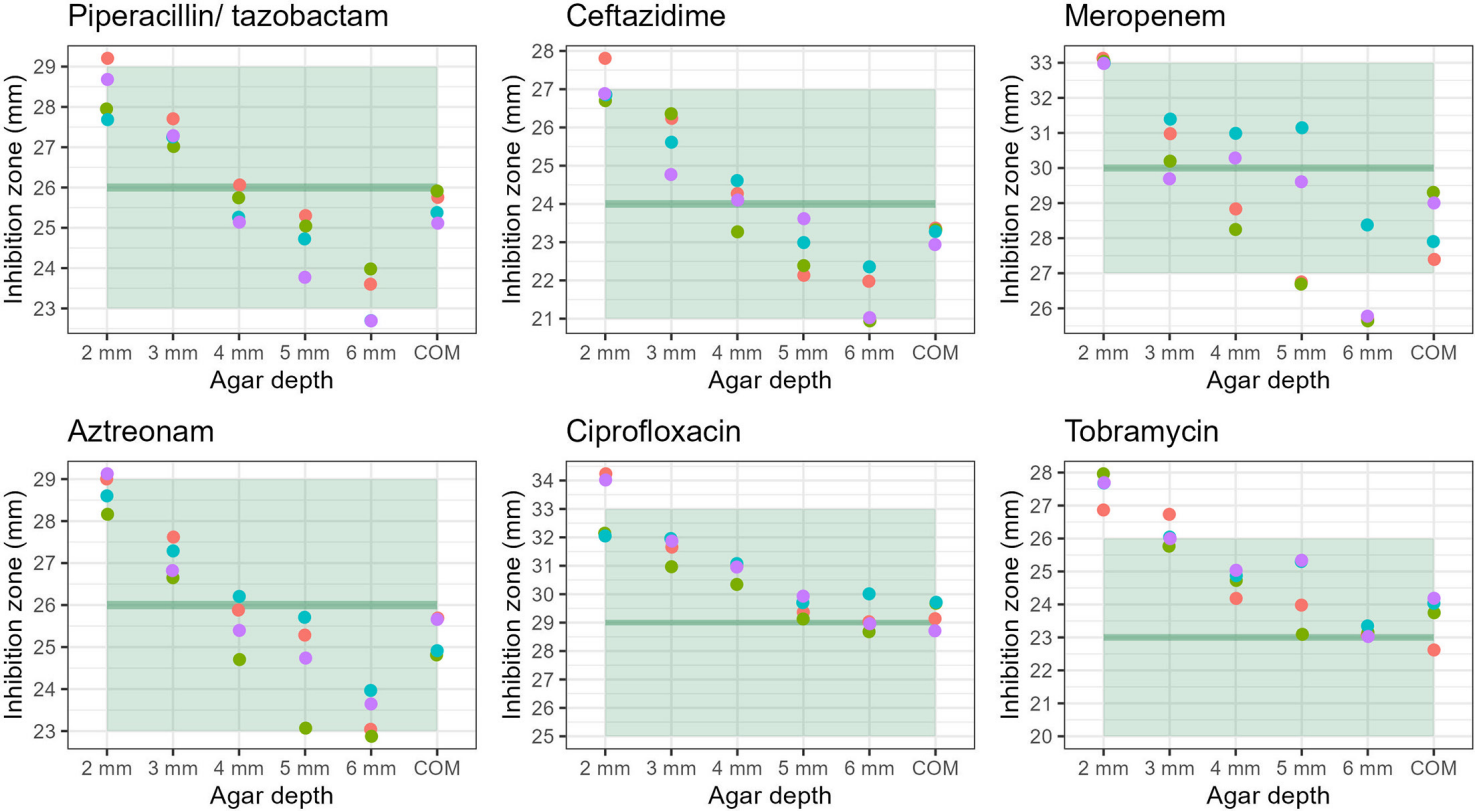
Comparison of *P. aeruginosa* ATCC 27853 inhibition zone diameters for the five agar depths of in-house MH agar and a commercial MH agar (COM) according to target (dark green) and range (light green).

**Fig. 3.**
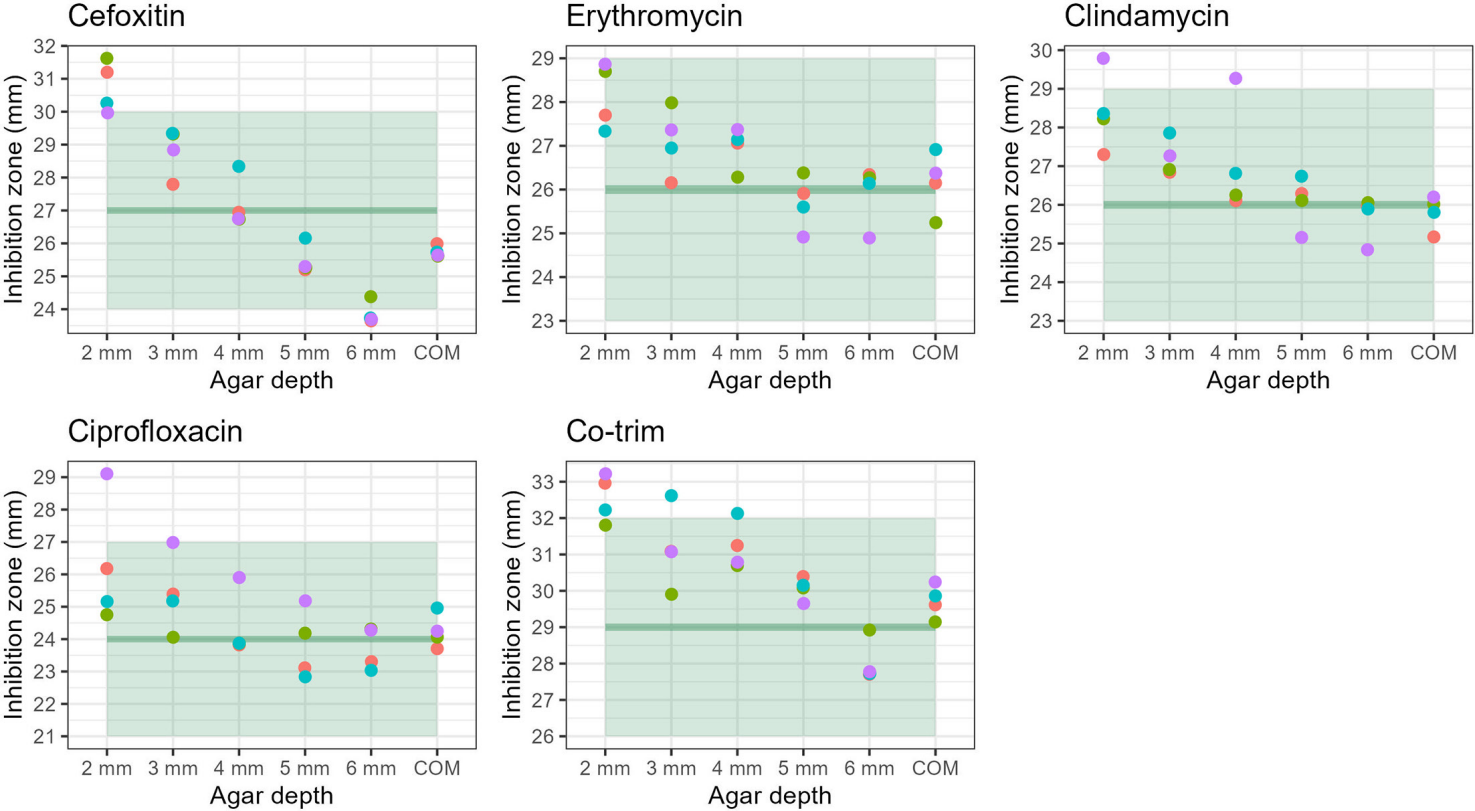
Comparison of *S. aureus* ATCC 29213 inhibition zone diameters for the five different agar depths of in-house MH agar and a commercial MH agar (COM) according to target (dark green) and range (light green).

The penalty scores were lowest for the commercial agar plates (0 to −4) followed by in-house 4-mm agar plates (−7 to −16). The penalty score increased the more the agar depth diverged from the standard (4 mm, Table S8).

### Bacterial isolates from patients

Most *S. aureus* isolates were methicillin resistant (60%), and most Enterobacterales had ceftriaxone resistance (80–90%, Table S4).

Apart from the 2-mm agar, all other agars met the criteria for the CA (≥90%, Table 1). While all agars had an ME rate of <3%, none of the tested agars met the criteria of the VME rate of <3% (5.8–12.5%, Table 1). The main reason for the high VME rates for all agar depths were the high proportions of false susceptibility to trimethoprim/sulfamethoxazole in *S. aureus* (up to 50%, Table S1) and high proportion of false susceptibility to ceftriaxone in Enterobacterales (up to 20% VME, Table S3). However, most zone diameters associated with VME for trimethoprim/sulfamethoxazole and *S. aureus* and for beta-lactam/beta-lactamase inhibitors and ceftriaxone for Enterobacterales were within 2 mm of the breakpoint (Tables S1–S3). Noteworthy, the cefoxitin screen had a CA of 100% independent of the agar depths (Table S1).

## Discussion

We tested various depths of in-house MH II agar and found that the inhibition zone diameters of QC strains are often within the acceptance range of tested antimicrobial agents. The accuracy of AST using different agar depths was hampered by a high proportion of VME while meeting the criteria for the CA and ME.

When considering the penalty scores [3] for QC strains, in-house media consistently performed less well than the commercial media, which had scores approaching zero. However, scores for the in-house agar at optimal depths were close to those reported for well-performing commercial media [3].

Although agar depth of 3–6 mm met the criteria of a CA ≥90% for bacterial isolates from patients, none of them had VME <3% (Table 1). Even the 4-mm in-house agar plates, the recommended depth did not fulfil a VME <3% in our approach when using patient isolates. These findings may be due to other factors that can impact on AST, such as pH, cation and thymidine content [8].

The continuous decrease in VME with increasing agar depth (Table 1) is likely due to the increase in the agar volume: the deeper the agar, the smaller the inhibition zone and the lower the false susceptibility rate (VME; Table 1) [2]. Particularly, the VME rate in *S. aureus* and Enterobacterales for trimethoprim/sulfamethoxazole was high, which is in line with recent observations in both pre-poured and in-house produced MH II agar [3,9].

Our study has several limitations. First, we only tested a small number of isolates from one setting impacting the diversity in resistance mechanisms and antimicrobial resistance patterns particularly for Enterobacterales. Second, we did not measure the pH of the media as recommended by the EUCAST [8], which might have affected the inhibition zone diameters. This was omitted to best reflect the real-life setting in resource-limited settings. Last, the accuracy of AST may have been affected by the choice of antimicrobials selected for testing.

In conclusion, the growth inhibition was usually within the acceptable ranges when using ATCC strains at optimum medium depth, and the diameter of the inhibition zone was correlated with culture medium depth. However, when using clinical isolates, VME was unacceptably high, particularly for agar depths that were not as recommended. This emphasizes the importance of ensuring optimal agar depth when preparing culture media in-house to prevent erroneous AST results that might impact patient care.

## Availability of data and materials

All data are made available in the manuscript and supplementary materials. Further requests can be made to the corresponding author.

## supplementary material

10.1099/jmm.0.001890 Uncited Table S1.

## Abbreviations

AST: antimicrobial susceptibility testing
BMD: broth microdilution
CA: categorical agreement
EUCAST: European Committee on Antimicrobial Susceptibility Testing
ME: major error
MH: Mueller–Hinton
MH II: Mueller–Hinton II
QC: quality control
VME: very major error
