# Supplementary material for: The impact of agar depth on antimicrobial susceptibility testing by disc diffusion
Source: J Med Microbiol. 2024 Sep 18;73(9):001890. doi: 10.1099/jmm.0.001890 (PMC11410041; doi:10.1099/jmm.0.001890)
Supplement: Uncited Table S1. [file jmm-73-01890-s001.pdf]

Supplement

Table S1: Evaluation of different depths of Mueller-Hinton II agar for the antimicrobial susceptibility testing of *Staphylococcus aureus*

Depth	Antimicrobial agent (disk content)	Categorical agreement [% (n/n_{total})]	Major errors [% (n/n_{total})]	Very Major errors [% (n/n_{total})]
2 mm	Trimethoprim/ sulfamethoxazole (1.25/23.75 µg)	50% (5/10)	0% (0/10)	50% (5/10)
	Cefoxitin-Screen (30 mg)	100% (10/10)	0% (0/10)	0% (0/10)
	Levofloxacin (5 µg)	100% (10/10)	0% (0/10)	0% (0/10)
	Clindamycin (2 µg)	100% (10/10)	0% (0/10)	0% (0/10)
	Rifampicin (5 µg)	60% (6/10)	40% (4/10)	0% (0/10)
	Linezolid (10 µg)	100% (10/10)	0% (0/10)	0% (0/10)
	**Total**	**85% (51/60)**	**7% (4/60)**	**8% (5/60)**
3 mm	Trimethoprim/ sulfamethoxazole (1.25/23.75 µg)	70% (7/10)	0% (0/10)	30% (3/10)
	Cefoxitin-Screen (30 mg)	100% (10/10)	0% (0/10)	0% (0/10)
	Levofloxacin (5 µg)	100% (10/10)	0% (0/10)	0% (0/10)
	Clindamycin (2 µg)	100% (10/10)	0% (0/10)	0% (0/10)
	Rifampicin (5 µg)	90% (9/10)	10% (1/10)	0% (0/10)
	Linezolid (10 µg)	100% (10/10)	0% (0/10)	0% (0/10)
	**Total**	**93% (56/60)**	**2% (1/60)**	**5% (3/60)**
4 mm	Trimethoprim/ sulfamethoxazole (1.25/23.75 µg)	80% (8/10)	0% (0/10)	20% (2/10)
	Cefoxitin-Screen (30 mg)	100% (10/10)	0% (0/10)	0% (0/10)
	Levofloxacin (5 µg)	100% (10/10)	0% (0/10)	0% (0/10)
	Clindamycin (2 µg)	100% (10/10)	0% (0/10)	0% (0/10)
	Rifampicin (5 µg)	80% (8/10)	20% (2/10)	0% (0/10)
	Linezolid (10 µg)	100% (10/10)	0% (0/10)	0% (0/10)
	**Total**	**93% (56/60)**	**3% (2/60)**	**3% (2/60)**
5 mm	Trimethoprim/ sulfamethoxazole (1.25/23.75 µg)	80% (8/10)	0% (0/10)	20% (2/10)
	Cefoxitin-Screen (30 mg)	100% (10/10)	0% (0/10)	0% (0/10)
	Levofloxacin (5 µg)	100% (10/10)	0% (0/10)	0% (0/10)
	Clindamycin (2 µg)	100% (10/10)	0% (0/10)	0% (0/10)
	Rifampicin (5 µg)	100% (10/10)	0% (0/10)	0% (0/10)
	Linezolid (10 µg)	100% (10/10)	0% (0/10)	0% (0/10)
	**Total**	**97% (58/60)**	**0% (0/60)**	**3% (2/60)**
6 mm	Trimethoprim/ sulfamethoxazole (1.25/23.75 µg)	90% (9/10)	0% (0/10)	10% (1/10)
	Cefoxitin-Screen (30 mg)	100% (10/10)	0% (0/10)	0% (0/10)
	Levofloxacin (5 µg)	100% (10/10)	0% (0/10)	0% (0/10)
	Clindamycin (2 µg)	100% (10/10)	0% (0/10)	0% (0/10)

	Rifampicin (5 µg)	100% (10/10)	0% (0/10)	0% (0/10)
	Linezolid (10 µg)	100% (10/10)	0% (0/10)	0% (0/10)
	**Total**	**98% (59/60)**	**0% (0/60)**	**2% (1/60)**

VME (disc diffusion S and Vitek R): trimethoprim/ sulfamethoxazole: most isolates (8/13) were within 2 mm of the breakpoint for S (17 mm).

Table S2: Evaluation of different depths of Mueller-Hinton II agar for the antimicrobial susceptibility testing of *Pseudomonas aeruginosa* (n=5)

Depth	Antimicrobial agent (disk content)	Categorical agreement [% (n/n_{total})]	Major errors [% (n/n_{total})]	Very Major errors [% (n/n_{total})]
2 mm	Piperacilliin/tazobactam (30/6 µg)	100% (5/5)	0% (0/5)	0% (0/5)
	Ceftazidim (10 µg)	100% (5/5)	0% (0/5)	0% (0/5)
	Ciprofloxacin (5 µg)	100% (5/5)	0% (0/5)	0% (0/5)
	Meropenem (10 µg)[a]	100% (5/5)	0% (0/5)	0% (0/5)
	Aztreonam (30 µg)	100% (5/5)	0% (0/5)	0% (0/5)
	Tobramycin (10 µg)	100% (5/5)	0% (0/5)	0% (0/5)
	**Total**	**100% (30/30)**	**0% (0/30)**	**0% (0/30)**
3 mm	Piperacilliin/tazobactam (30/6 µg)	100% (5/5)	0% (0/5)	0% (0/5)
	Ceftazidim (10 µg)	100% (5/5)	0% (0/5)	0% (0/5)
	Ciprofloxacin (5 µg)	100% (5/5)	0% (0/5)	0% (0/5)
	Meropenem (10 µg)[a]	100% (5/5)	0% (0/5)	0% (0/5)
	Aztreonam (30 µg)	100% (5/5)	0% (0/5)	0% (0/5)
	Tobramycin (10 µg)	100% (5/5)	0% (0/5)	0% (0/5)
	**Total**	**100% (30/30)**	**0% (0/30)**	**0% (0/30)**
4 mm	Piperacilliin/tazobactam (30/6 µg)	100% (5/5)	0% (0/5)	0% (0/5)
	Ceftazidim (10 µg)	100% (5/5)	0% (0/5)	0% (0/5)
	Ciprofloxacin (5 µg)	80% (4/5)	20% (1/5)	0% (0/5)
	Meropenem (10 µg)[a]	100% (5/5)	0% (0/5)	0% (0/5)
	Aztreonam (30 µg)	100% (5/5)	0% (0/5)	0% (0/5)
	Tobramycin (10 µg)	100% (5/5)	0% (0/5)	0% (0/5)
	**Total**	**97% (29/30)**	**3% (1/30)**	**0% (0/30)**
5 mm	Piperacilliin/tazobactam (30/6 µg)	100% (5/5)	0% (0/5)	0% (0/5)
	Ceftazidim (10 µg)	100% (5/5)	0% (0/5)	0% (0/5)
	Ciprofloxacin (5 µg)	80% (4/5)	20% (1/5)	0% (0/5)
	Meropenem (10 µg)[a]	100% (5/5)	0% (0/5)	0% (0/5)
	Aztreonam (30 µg)	100% (5/5)	0% (0/5)	0% (0/5)
	Tobramycin (10 µg)	100% (5/5)	0% (0/5)	0% (0/5)
	**Total**	**97% (29/30)**	**3% (1/30)**	**0% (0/30)**
6 mm	Piperacilliin/tazobactam (30/6 µg)	100% (5/5)	0% (0/5)	0% (0/5)
	Ceftazidim (10 µg)	100% (5/5)	0% (0/5)	0% (0/5)
	Ciprofloxacin (5 µg)	80% (4/5)	20% (1/5)	0% (0/5)
	Meropenem (10 µg)[a]	100% (5/5)	0% (0/5)	0% (0/5)
	Aztreonam (30 µg)	100% (5/5)	0% (0/5)	0% (0/5)
	Tobramycin (10 µg)	100% (5/5)	0% (0/5)	0% (0/5)
	**Total**	**97% (29/30)**	**3% (1/30)**	**0% (0/30)**

[a]meningitis-breakpoints, EUCAST, Version 13.1

VME (disc diffusion S and MIC-based method R): none

Table S3: Evaluation of different depths of Mueller-Hinton II agar for the antimicrobial susceptibility testing of *Escherichia coli* (n=10) and *Klebsiella pneumoniae* (n=10)

Depth	Antimicrobial agent (disk content)	Categorical agreement [% (n/n_{total})]	Major errors [% (n/n_{total})]	Very Major errors [% (n/n_{total})]
2 mm	Ampicillin (10 µg)	95% (19/20)	0% (0/20)	5% (1/20)
	Ampicillin/sulbactam (10/10 µg)	60% (12/20)	0% (0/20)	40% (8/20)
	Piperacillin/tazobactam (30/6 µg)	65% (13/20)	0% (0/20)	35% (7/20)
	Ceftriaxone (30 µg)[a]	80% (16/20)	0% (0/20)	20% (4/20)
	Meropenem (10 µg)[a]	100% (20/20)	0% (0/20)	0% (0/20)
	Ciprofloxacin (5 µg)[b]	85% (17/20)	0% (0/20)	15% (3/20)
	Tigecyclin (15 mg)[c]	100% (10/10)	0% (0/10)	0% (0/10)
	Trimethoprim/ sulfamethoxazole (1.25/23.75 µg)	90% (18/20)	0% (0/20)	10% (2/20)
	**Total**	**83% (125/150)**	**0% (0/150)**	**17% (25/150)**
3 mm	Ampicillin (10 µg)	100% (20/20)	0% (0/20)	0% (0/20)
	Ampicillin/sulbactam (10/10 µg)	80% (16/20)	0% (0/20)	20% (4/20)
	Piperacillin/tazobactam (30/6 µg)	85% (17/20)	0% (0/20)	15% (3/20)
	Ceftriaxone (30 µg)[a]	85% (17/20)	0% (0/20)	15% (3/20)
	Meropenem (10 µg)[a]	100% (20/20)	0% (0/20)	0% (0/20)
	Ciprofloxacin (5 µg)[b]	95% (19/20)	0% (0/20)	5% (1/20)
	Tigecyclin (15 mg)[c]	100% (10/10)	0% (0/10)	0% (0/10)
	Trimethoprim/ sulfamethoxazole (1.25/23.75 µg)	90% (18/20)	0% (0/20)	10% (2/20)
	**Total**	**91% (137/150)**	**0% (0/150)**	**9% (13/150)**
4 mm	Ampicillin (10 µg)	95% (19/20)	0% (0/20)	5% (1/20)
	Ampicillin/sulbactam (10/10 µg)	85% (17/20)	0% (0/20)	15% (3/20)
	Piperacillin/tazobactam (30/6 µg)	90% (18/20)	0% (0/20)	10% (2/20)
	Ceftriaxone (30 µg)[a]	90% (18/20)	0% (0/20)	10% (2/20)
	Meropenem (10 µg)[a]	100% (20/20)	0% (0/20)	0% (0/20)
	Ciprofloxacin (5 µg)[b]	95% (19/20)	0% (0/20)	5% (1/20)
	Tigecyclin (15 mg)[c]	100% (10/10)	0% (0/10)	0% (0/10)
	Trimethoprim/ sulfamethoxazole (1.25/23.75 µg)	90% (18/20)	0% (0/20)	10% (2/20)
	**Total**	**93% (139/150)**	**0% (0/150)**	**7% (11/150)**
5 mm	Ampicillin (10 µg)	90% (18/20)	10% (2/20)	0% (0/20)
	Ampicillin/sulbactam (10/10 µg)	85% (17/20)	0% (0/20)	15% (3/20)
	Piperacillin/tazobactam (30/6 µg)	100% (20/20)	0% (0/20)	0% (0/20)

	Ceftriaxone (30 µg)[a]	85% (17/20)	0% (0/20)	15% (3/20)
	Meropenem (10 µg)[a]	100% (20/20)	0% (0/20)	0% (0/20)
	Ciprofloxacin (5 µg)[b]	90% (18/20)	0% (0/20)	10% (2/20)
	Tigecyclin (15 mg)[c]	100% (10/10)	0% (0/10)	0% (0/10)
	Trimethoprim/ sulfamethoxazole (1.25/23.75 µg)	90% (18/20)	0% (0/20)	10% (2/20)
	**Total**	**92% (138/150)**	**1% (2/150)**	**7% (10/150)**
6 mm	Ampicillin (10 µg)	90% (18/20)	10% (2/20)	0% (0/20)
	Ampicillin/sulbactam (10/10 µg)	90% (18/20)	0% (0/20)	10% (2/20)
	Piperacillin/tazobactam (30/6 µg)	90% (18/20)	10% (2/20)	0% (0/20)
	Ceftriaxone (30 µg)[a]	90% (18/20)	0% (0/20)	10% (2/20)
	Meropenem (10 µg)[a]	100% (20/20)	0% (0/20)	0% (0/20)
	Ciprofloxacin (5 µg)[b]	95% (19/20)	0% (0/20)	5% (1/20)
	Tigecyclin (15 mg)[c]	100% (10/10)	0% (0/10)	0% (0/10)
	Trimethoprim/ sulfamethoxazole (1.25/23.75 µg)	90% (18/20)	0% (0/20)	10% (2/20)
	**Total**	**93% (139/150)**	**3% (4/150)**	**5% (7/150)**

[a]meningitis-breakpoints, EUCAST, Version 13.1

[b]non-meningitis breakpoints, EUCAST, Version 13.1

[c]for *Escherichia coli* only

VME (disc diffusion S and MIC-based method R): ampicillin: 0/2 tests within 2 mm of breakpoint; ampicillin/ sulbactam: 8/19 tests were within 2 mm of the breakpoint; piperacillin/ tazobactam: 12/12 isolates were within 2 mm of the breakpoint; ceftriaxone: 13/14 tests were within 2 mm of the breakpoint for S (non-meningitis); ciprofloxacin: 1/8 tests were within 2 mm of the breakpoint for S and 2/8 were interpreted as I; trimethoprim/ sulfamethoxazole: 0/10 were within 2 mm of the breakpoint.

Table S4: Antimicrobial resistance of isolates from Sierra Leone using the reference method

Antimicrobial agent	*Staphylococcus aureus* (n=10)	*Pseudomonas aeruginosa* (n=5)	*Escherichia coli* (n=10)	*Klebsiella pneumoniae* (n=10)
Ampicillin	-	-	80% (8)	100% (10)
Ampicillin/sulbactam	-	-	70% (7)	80% (8)
Piperacillin/tazobactam	-	0% (0)	40% /4)	50% (5)
Cefoxitin	60% (6)	-	-	-
Ceftriaxone[a]	-	-	90% (9)	80% (8)
Ceftazidim	-	0% (0)	-	-
Meropenem	-	0% (0)	0% (0)	0% (0)
Trimethoprim/ sulfamethoxazole	70% (7)	-	90% (9)	70% (7)
Ciprofloxacin	-	0% (0)	80% (8)	60% (6)
Levofloxacin	60% (6)	-	-	-
Clindamycin	40% (4)	-	-	-
Rifampicin	0% (0)	-	-	-
Linezolid	0% (0)	-	-	-
Aztreonam	-	0% (0)	-	-
Tobramycin	-	20% (1)	-	-
Tigecyclin (15 mg)	-	-	0% (0)	-

% (n resistant isolates) according to broth microdilution or Vitek2 automated systems,
- = not applicable

[a]meningitis-breakpoints, EUCAST, Version 13.1

Table S5: Mean zone of inhibition according to agar depth and media type for ATCC 25922 *Escherichia coli*

			Mean zone of inhibition [mm] at various MH agar plates					
	**Target**	**Range**	**2 mm**	**3 mm**	**4 mm**	**5 mm**	**6 mm**	**Commercial**
Ampicillin	18-19	15-22	23*	21	20	18	17	19#
Ampicillin/ sulbactam	21-22	19-24	25*	23	22#	20	19	21#
Piperacillin	24	21-27	27	26	25	23	23	24#
Piperacillin/ tazobactam	24	21-27	27*	25	25	24	23	24#
Ceftriaxone	32	29-35	33	32	32#	30	29	31
Imipenem	29	26-32	32*	31	30	29	28	29#
Meropenem	31-32	28-35	35*	33	33	32	31	31#
Ciprofloxacin	33	29-37	38*	37*	36	35	34	34
Gentamicin	22-23	19-26	27*	26*	25	25	24	23#
Co-trimoxazole	26	23-29	29*	28	27	25	24	26#
Fosfomycin	30	26-34	33	31	29	28	28	30#
Tigecycline	23-24	20-27	25	24	23#	22	22	23#

*≥1 replicate value outside the accepted range

mean value for ideal depth on target

Table S6: Mean zone of inhibition according to agar depth and media type for *Pseudomonas aeruginosa* ATCC 27853

			Mean zone of inhibition at various MH agar plates					
	**Target**	**Range**	**2 mm**	**3 mm**	**4 mm**	**5 mm**	**6 mm**	**Commercial**
Piperacillin/ tazobactam	26	23-29	29	27	26#	25	24	26#
Ceftazidime	24	21-27	27*	26	24#	23	22	23
Meropenem	30	27-33	33	31	30#	29	27*	28
Aztreonam	26	23-29	29	27	26#	25	24	26#
Ciprofloxacin	29	25-33	33*	32	31	30	29	30
Tobramycin	23	20-26	28*	26*	25	24	23	24

*≥1 replicate value outside the accepted range

mean value for ideal depth on target

Table S7: Mean zone of inhibition according to agar depth and media type for *Staphylococcus aureus* ATCC 29213

			Mean zone of inhibition at various MH agar plates					
	**Target**	**Range**	**2 mm**	**3 mm**	**4 mm**	**5 mm**	**6 mm**	**Commercial**
Cefoxitin	27	24-30	31*	29	27#	25	24	26
Erythromycin	26	23-29	28	27	27	26	26	26#
Clindamycin	26	23-29	28*	27	27	26	26	26#
Ciprofloxacin	24	21-27	26*	25	25	24	24	24#
Co-trimoxazole	29	26-32	33*	31*	31	30	28	30

*≥1 replicate value outside the accepted range

mean value for ideal depth on target

Table S8: Penalty scores and performance of different agar depths using ATCC strains

	2 mm	3 mm	4 mm	5 mm	6 mm	Commercial
E. coli ATCC 25922						
Penalty score	-132	-47	-16	-21	-41	-1
Proportion within ±1 of target	17%	56%	75%	73%	60%	98%
Proportion out of range	27%	6%	0%	0%	0%	0%
P. aeruginosa ATCC 27853						
Penalty score	-80	-29	-7	-14	-80	-4
Proportion within ±1 of target	0%	46%	71%	67%	0%	92%
Proportion out of range	29%	4%	0%	0%	46%	0%
S. aureus ATCC 29213						
Penalty score	-52	-15	-10	-3	-72	0
Proportion within ±1 of target	20%	55%	70%	85%	20%	100%
Proportion out of range	30%	5%	0%	0%	60%	0%

Penalty scores were calculated as follows: inhibition zone on target ±1 mm (0 points); inhibition zone within ±2 mm but more than ±1 mm (-1 points); inhibition zone within ±3 mm but more than ±2 mm and within range (-3 points); inhibition zone outside range (-5 points). The minimum score was 0 and the maximum -240 (quadruplicate for 12 antibiotics multiplied by -5) for E. coli, -120 for P. aeruginosa (quadruplicate for 6 antibiotics) and -100 for S. aureus (quadruplicate for 5 antibiotics). The denominator for the proportions was total antibiotics tested in quadruplicate: 48 for E. coli, 24 for P. aeruginosa.[1]

References

1. Ahman J, Matuschek E, Kahlmeter G. EUCAST evaluation of 21 brands of Mueller-Hinton dehydrated media for disc diffusion testing. *Clin Microbiol Infect* 2020; **26**(10): 1412 e1- e5.
